# Supplementary material for: Multiple ancestral haplotypes harboring regulatory mutations cumulatively contribute to a QTL affecting chicken growth traits
Source: Commun Biol. 2020 Aug 28;3:472. doi: 10.1038/s42003-020-01199-3 (PMC7455696; doi:10.1038/s42003-020-01199-3)
Supplement: Supplementary file 1 — Supplementary Information [file 42003_2020_1199_MOESM1_ESM.pdf]

Supplementary Information for “Multiple ancestral haplotypes harboring regulatory mutations cumulatively contribute to a QTL affecting chicken growth traits”

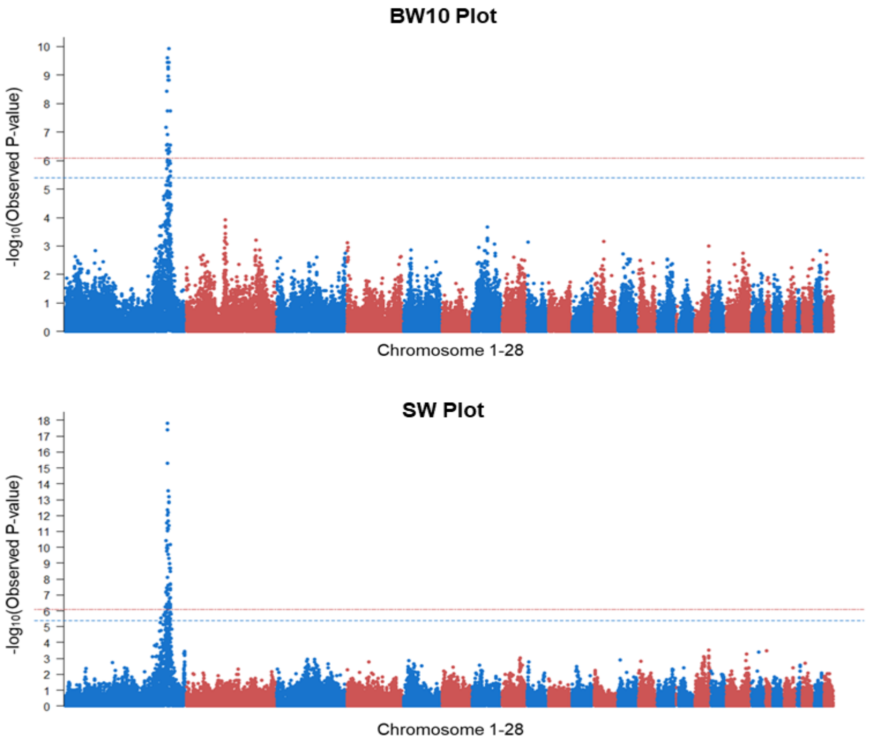

Supplementary Fig. 1. Manhattan plots for GWAS of BW10 and SW in F_2 generation. The GWAS results of BW10 and SW are displayed as $-\log_{10}(P)$ values at the chromosomal position of all genome-wide tested SNPs. The horizontal red/blue lines indicate the genome-wide 1/5% significance thresholds (P values = 8.12×10^{-7}/4.06×10^{-6}), respectively.

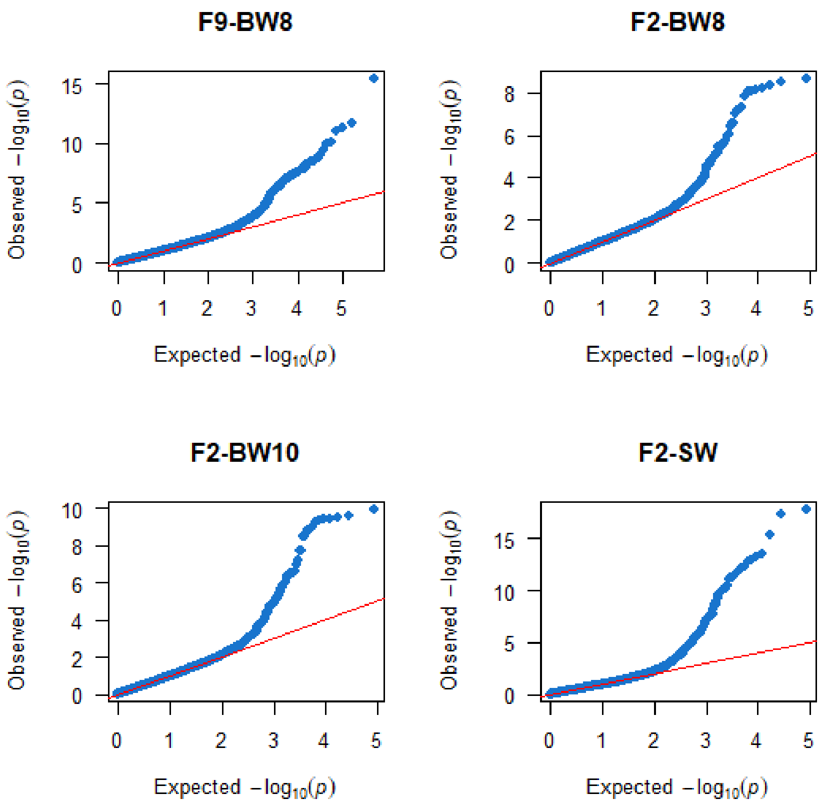

Supplementary Fig. 2. Q-Q plots for the results from the genome wide association analyses. The figures presented are for body weight (BW) and the combined weight of the ventriculus and the proventriculus (SW) in the F_2 and F_9 generations of the HB × HQLA deep intercross population.

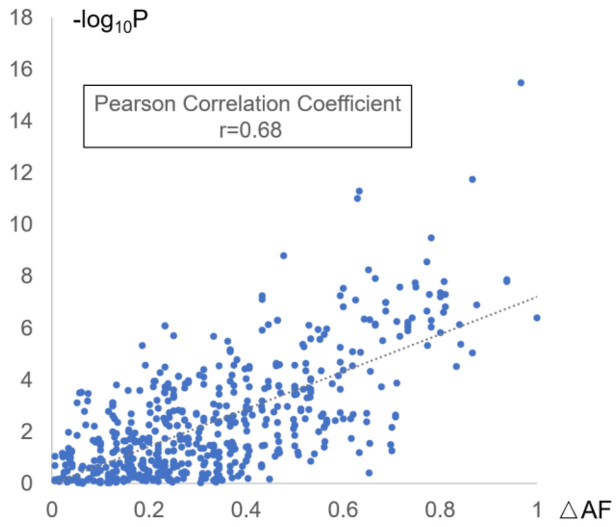

Supplementary Fig. 3. Relationship between the allele-frequency differences of all SNPs in this QTL and the P value of association to BW8. The △AF between the HQLA and HB are displayed as $-\log_{10}(P)$ values of 609 tested SNPs in 3.1 Mb QTL interval. The Pearson correlation coefficient was 0.68.

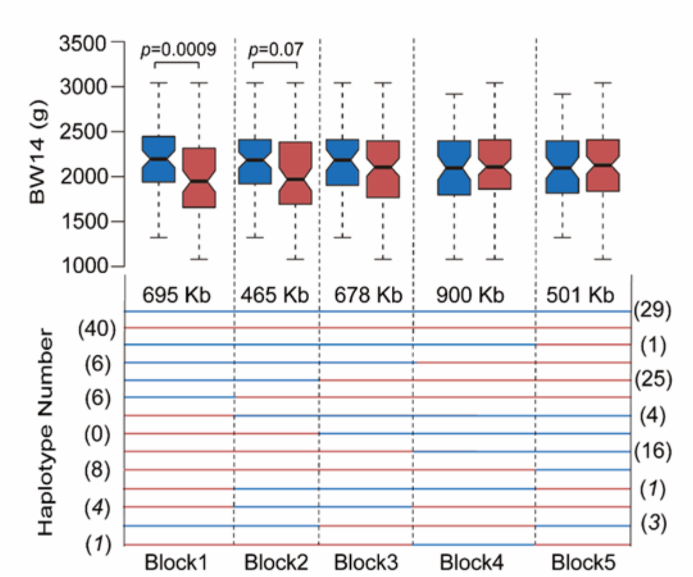

Supplementary Fig. 4. Results from IBD-based fine-mapping analysis of the association region on Chromosome 1. The top association region (GGA1 168.2-171.3 Mb) was fine-mapped using an IBD-based association analysis in the F_9 generation of the deep intercross population. Sub-regions IBD to HB and HQLA in the F_0 generation were identified. The seventy-two F_9 individuals (144 chromosomes) carried recombinant chromosomes from F_0 to F_9 were used in the analyses. Blue/red lines represent chromosome segments inherited from HQLA/HB, respectively. Significant association was found to block1 for body-weight at 2-weeks of age (BW14; P = 0.0009).

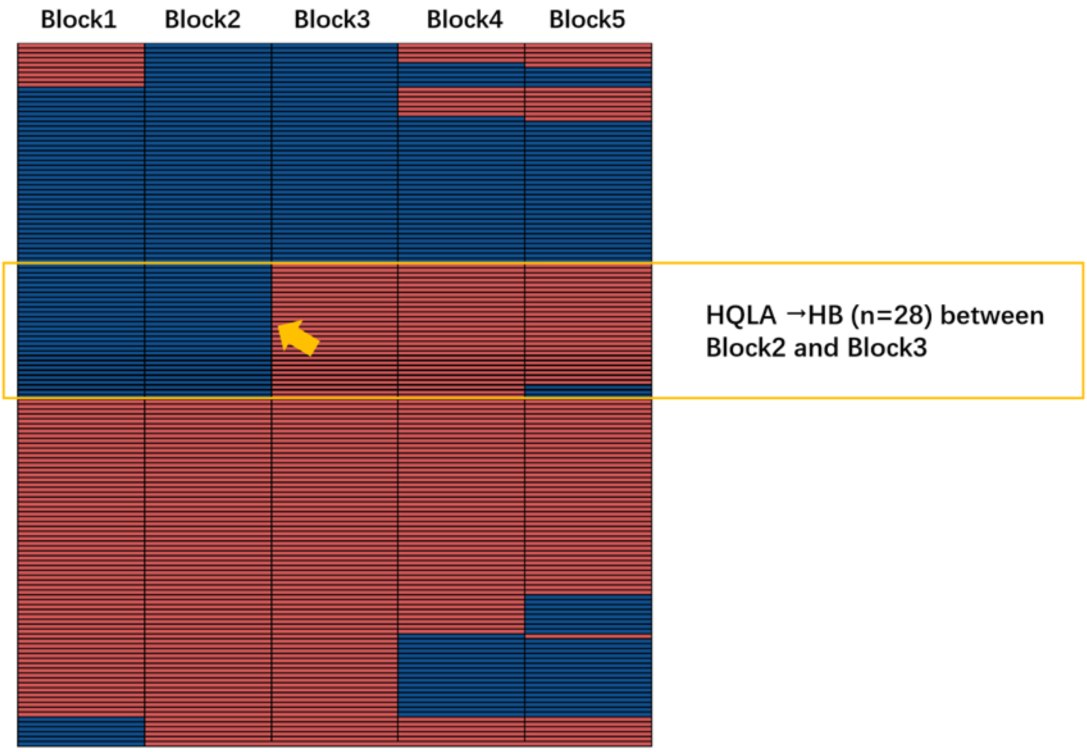

Supplementary Fig. 5. Illustration of the founder-breed haplotypes across the five blocks in the 72 individuals (144 chromatids) carrying recombinant chromosomes in the region from 168.8 to 171.7 Mb on Chromosome 1. Blue/red lines represent chromosome segments inherited from the HQLA/HB. The only difference between Block2 and Block3 is the 28 chromatids converted from HQLA to HB.

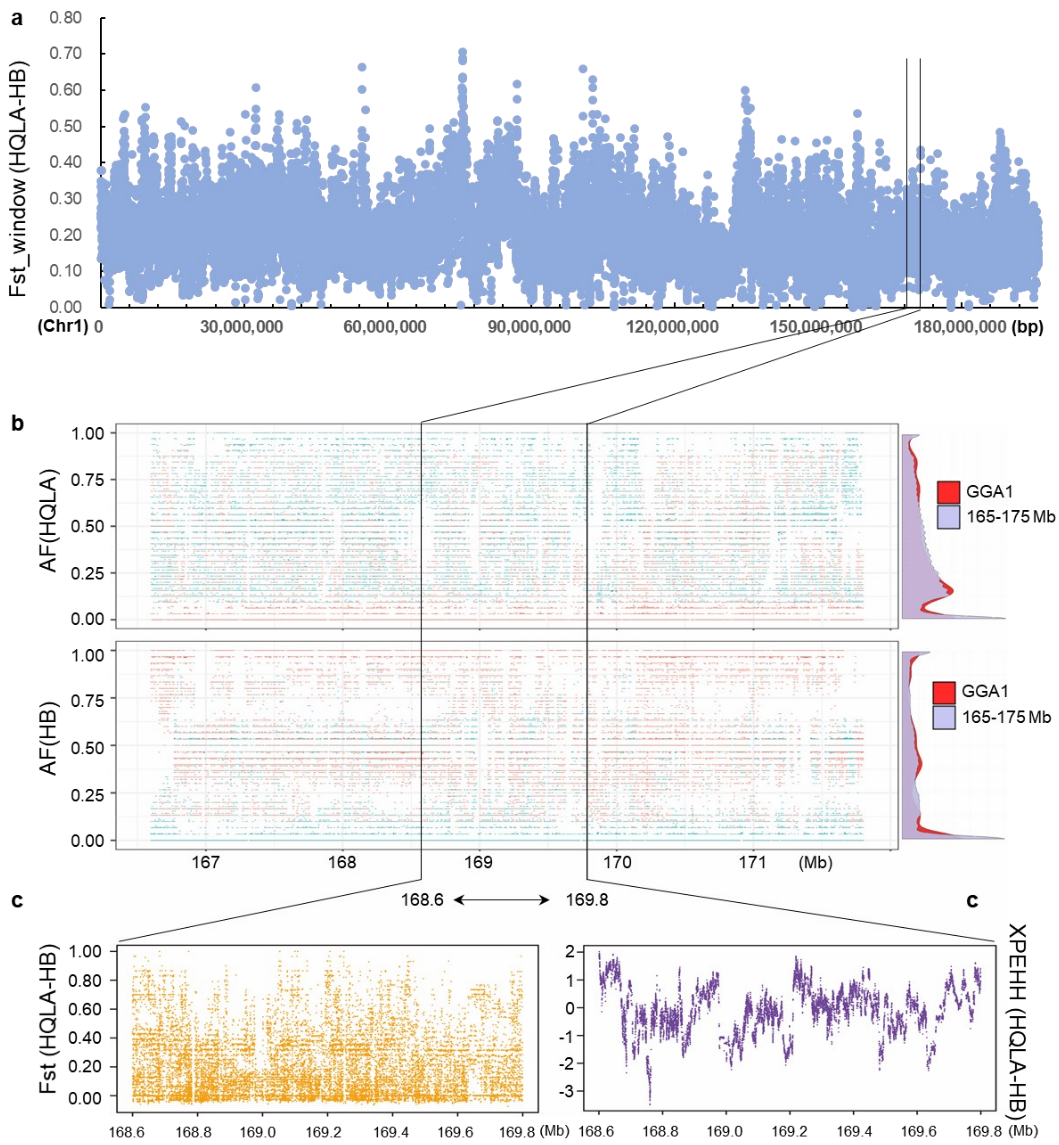

Supplementary Fig. 6. Mosaic signatures of selection in 1.2 Mb region. (a) Fst estimates between HQLA and HB on chromosome 1 in bins of 20 Kbp. **(b)** Allele frequency spectrum in the HQLA and HB stocks. SNPs with higher frequency in HQLA/HB are plotted in green/ orange, respectively. Density curves in red represent statistics on the entire GGA1 and gray blue in represent statistics on the GGA1: 165-175 Mb interval. **(c)** Fst estimates on a per-site basis and **c)** normalized XPEHH value between HQLA and HB. Scatter plots represent the fine-mapped 1.2 Mb interval (169.6-169.8 Mb).

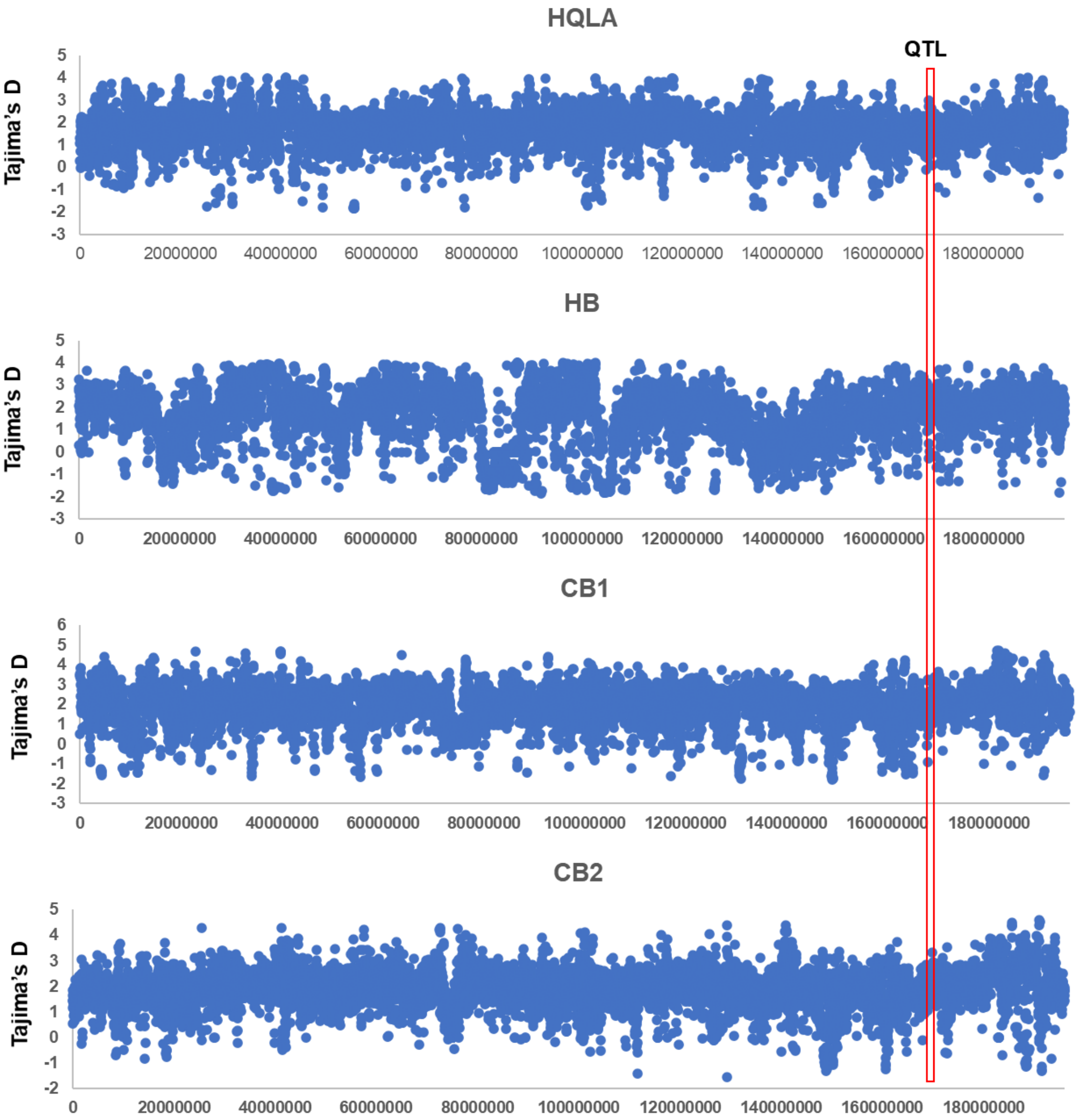

Supplementary Fig. 7. Tajima's D statistic in HQLA, HB, CB1, CB2 stocks on chromosome 1. Tajima's D statistic was calculated in bins with 25 Kbp.

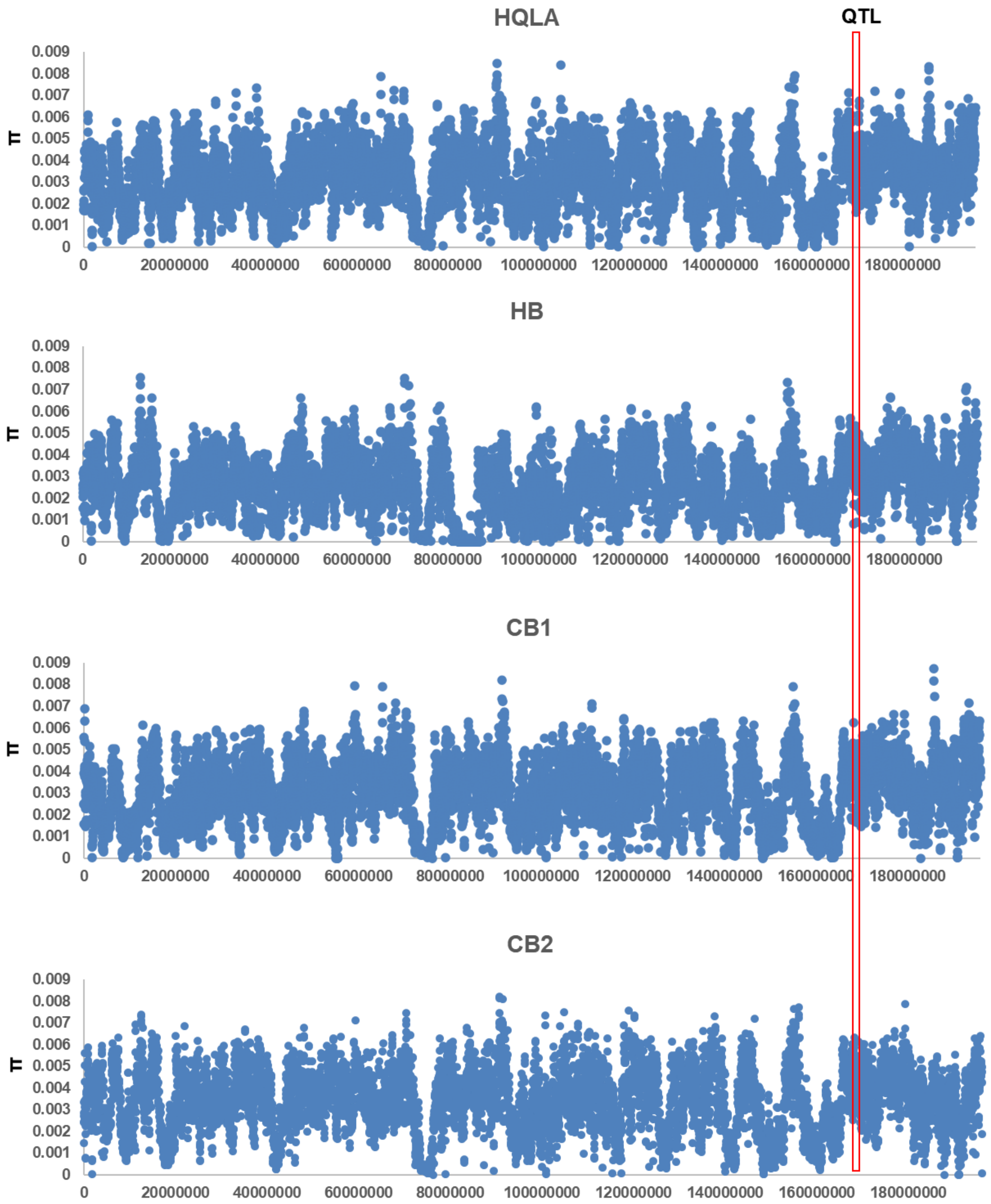

Supplementary Fig. 8. Nucleotide diversity (π) in HQLA, HB, CB1, CB2 stocks on chromosome 1. Nucleotide diversity (π) were measured in windows of 500 bp and a step size of 25 Kbp.

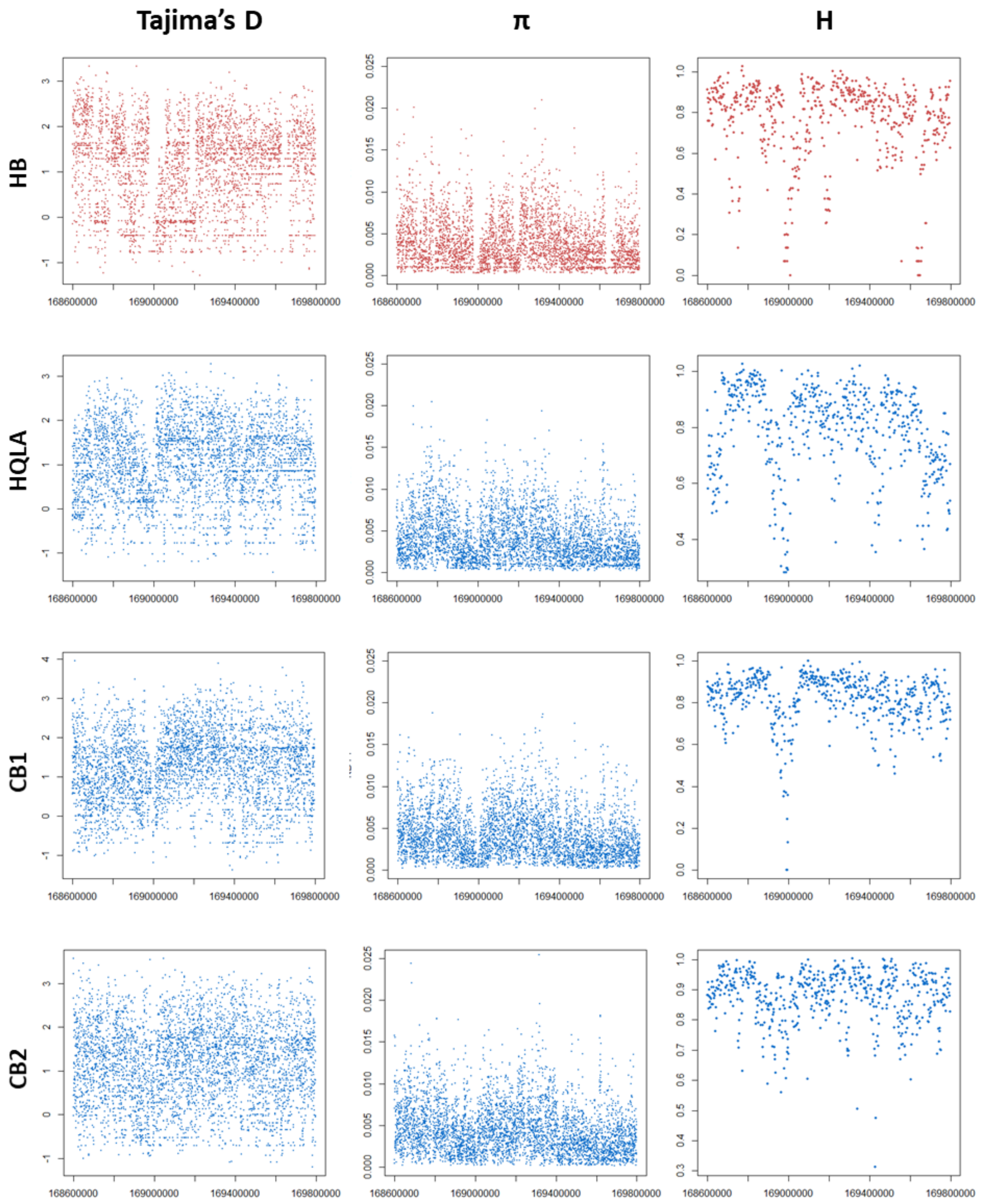

Supplementary Fig. 9. Genetic diversity in four chicken stocks across the fine-mapped 1.2 Mb region on Chromosome 1. Illustration of three measures of the genetic diversity (Tajima's D, π and haplotype diversity (H)) in the founders for the deep intercross (HB, HQLA) and two high-weight broiler breeds (CB1, CB2). The scatter plots represent the 1.2 Mb fine-mapped interval on GGA1 (168.6-169.8 Mb). Tajima's D statistic was calculated in bins with 250 bp. Nucleotide diversity (π) were measured in windows of 500 bp and a step size of 250 bp. The H statistic was calculated in bins of 2 Kbp.

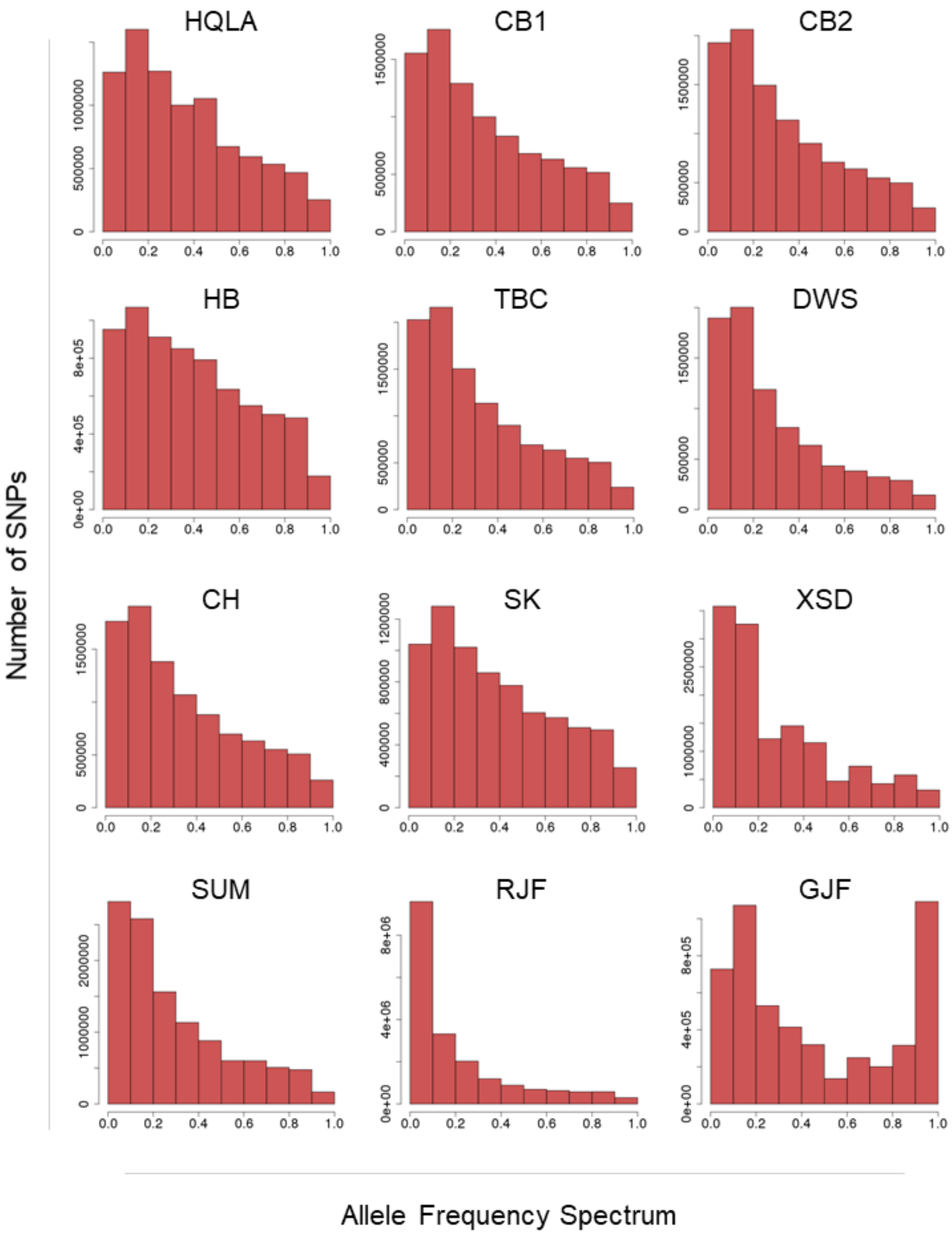

Supplementary Fig. 10. The genome-wide allele frequency spectrum across the 12 analyzed chicken breeds. The x-axis denotes the allele frequency-bins (steps of 0.1) and the y-axis the number of SNPs discovered by whole genome re-sequencing in each bin. HQLA: High quality line A03; CB1: Cornish; CB2: Recessive white Rock; HB: Huiyang Bearded chicken; TBC: Tibetan chicken; DWS: Daweishan Mini chicken; CH: Chahua chicken; SK: Silkies; XSD: Xishuangbanna Game chicken; SUM: Sumatera and Kedu Hitam; RJF: Red jungle fowl; GJF: Green jungle fowl.

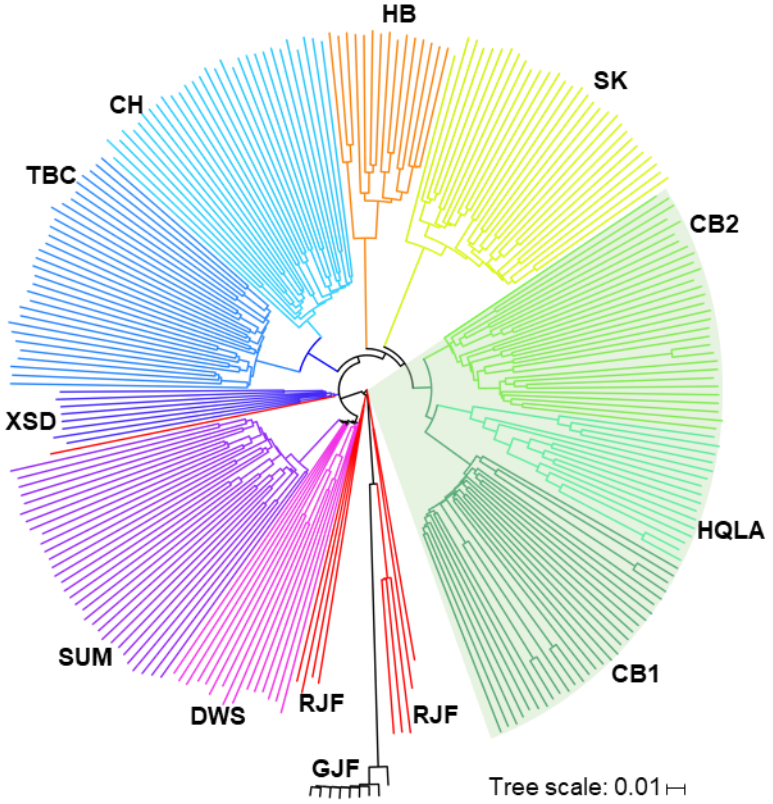

Supplementary Fig. 11. Neighbor Joining (NJ) cladogram of all 253 samples. Neighbor Joining (NJ) cladogram based on independent-pairwise genome-wide SNPs. Taxa represent the 253 samples assessed in this study, with fill color corresponding to population membership. HQLA: High quality line A03; CB1: Cornish; CB2: Recessive white Rock; HB: Huiyang Bearded chicken; TBC: Tibetan chicken; DWS: Daweishan Mini chicken; CH: Chahua chicken; SK: Silkies; XSD: Xishuangbanna Game chicken; SUM: Sumatera and Kedu Hitam; RJF: Red jungle fowl; GJF: Green jungle fowl.

	Q1	Q2	Q3	Q4	Q5	Q6	Q7	Q8	Q9
Q1	0.47	0.49	-0.16	-0.04	0.20	0.10	-0.07	-0.01	0.06
Q2	0.202	0.42	-0.18	0.07	0.12	-0.03	0.06	-0.04	0.11
Q3	0.01	0.016	0.75	0.01	-0.09	-0.18	0.14	-0.14	-0.26
Q4	0.002	0.003	6E-05	0.51	0.29	0.12	0.18	0.09	0.13
Q5	0.032	0.015	0.004	0.06	0.420	0.18	0.03	0.11	0.84
Q6	0.008	5E-04	0.008	0.011	0.020	0.55	0.44	0.33	0.26
Q7	0.003	0.003	0.005	0.023	9E-04	0.116	0.43	0.41	0.32
Q8	5E-05	1E-03	0.009	0.007	0.011	0.072	0.153	0.45	0.70
Q9	0.002	0.006	0.015	0.011	0.330	0.056	0.051	0.254	0.61

Supplementary Fig. 12. Illustration of the pairwise linkage disequilibrium (LD) of Q1-Q9 across 150 sequenced chromosomes in 75 high-weight (broiler) samples. The diagonal and lower/upper triangles give the haplotype frequencies of the segments Q1-Q9, pairwise D’ values and pairwise r^2 values, respectively.

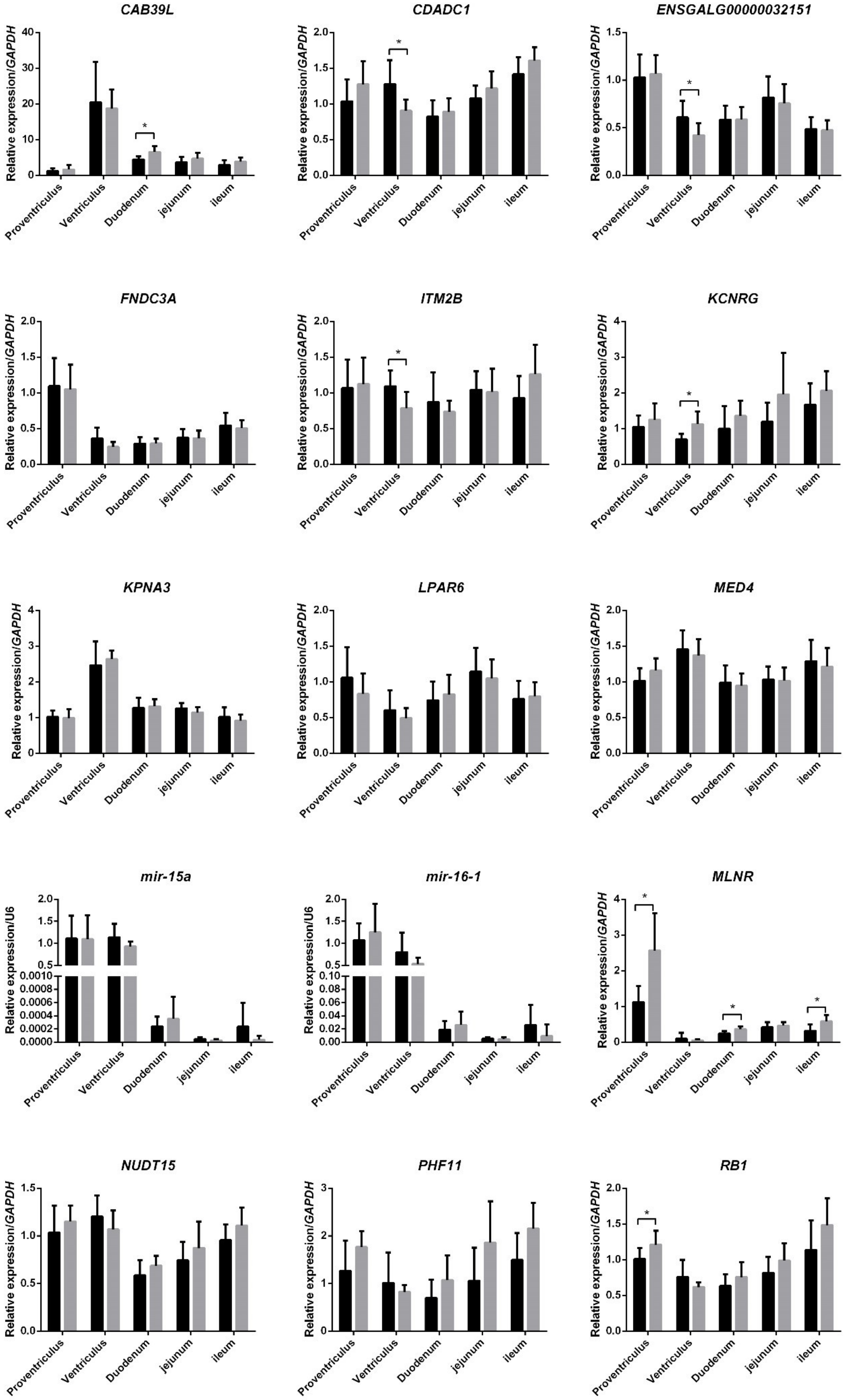
CAB39L
CDADC1
ENSGALG00000032151
FNDC3A
ITM2B
KCNRG
KPNA3
LPAR6
MED4
mir-15a
mir-16-1
MLNR
NUDT15
PHF11
RB1
Relative expression/GAPDH
Relative expression/U6
Proventriculus
Ventriculus
Duodenum
jejunum
ileum
*

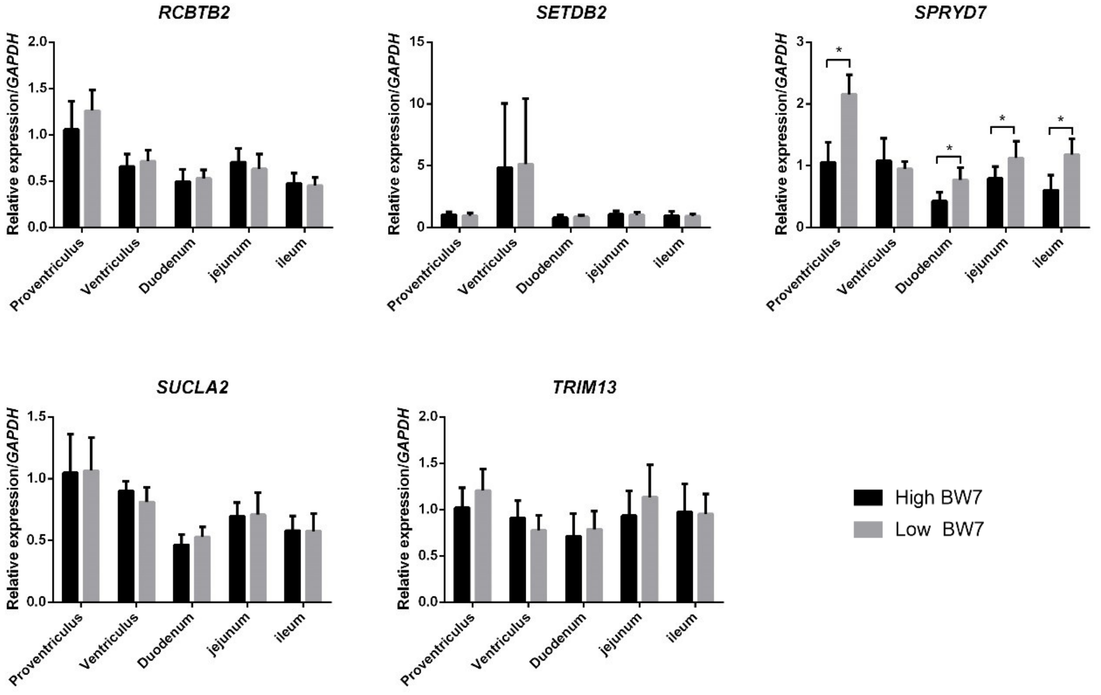

Supplementary Fig. 13. Results from the gene expression analyses of candidate genes within the QTL region. Samples were high and low 7-week body-weight chickens from the F_{15} generation of the deep intercross across multiple tissues. The data are presented as the means ± Std Error. Stars represent $p < 0.05$ that were estimated by Student's t-test.

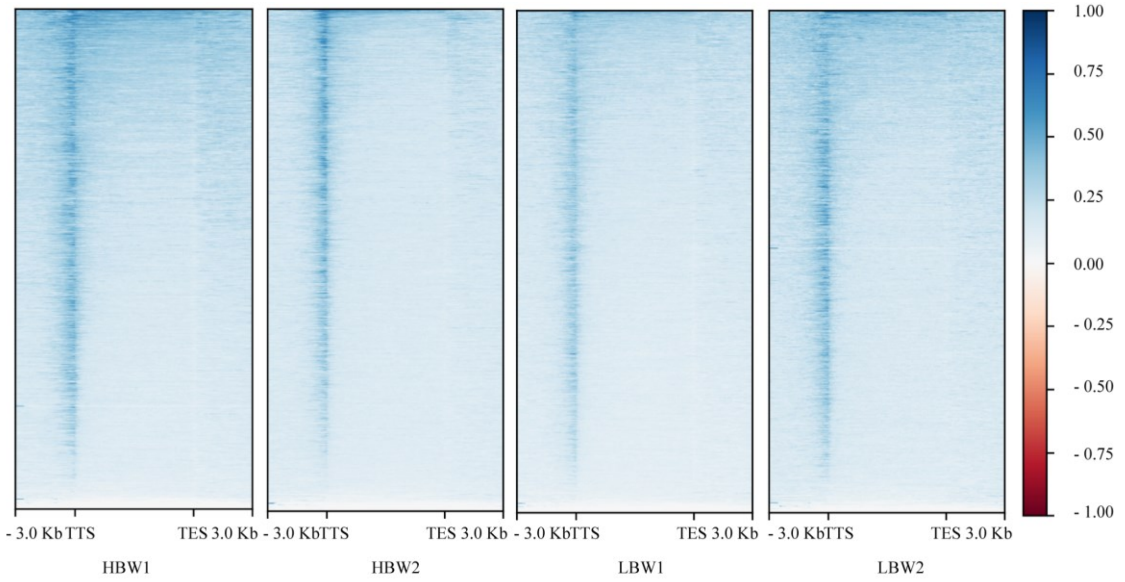

Supplementary Fig. 14. Peak distribution from the ATAC sequencing near coding regions. A characteristic enrichment near gene transcriptional start sites (TSS) for all samples.

Supplementary Table 1 Descriptive statistics for the HQLA-HB AIL pedigree

Generation	Nb of animals	Nb of males contributing to the next generation	Nb of females contributing to the next generation	Average body weight at 49 days of age (g) ± SE	Average body weight at 56 days of age (g) ± SE
**F0-HB**	196	4	12	——	545.26±5.48
**F0-HQLA**	156	4	12	——	1751.99±16.05
**F1**	414	8	48	——	1106.94± 7.75
**F2**	1627	72	72	954.52±5.56	1239.47± 7.15
**F3**	655	98	98	849.19±4.19	——
**F4**	1163	99	179	924.73±4.14	——
**F5**	1131	99	175	791.95±9.79	——
**F6**	1165	99	195	832.14±3.96	——
**F7**	1605	101	186	951.97±3.77	——
**F8**	1274	95	95	992.33±4.46	——
**F9**	1314	——	——	966.68±7.17	1144.20±7.78

Supplementary Table 2 Descriptive statistics of the phenotypes for F_9 individuals

Traits	n	mean	sd	min	max
**BW0**	587	30.17	2.85	21.5	39.8
**BW2**	589	166.63	17.98	99.6	219.3
**BW4**	585	433.93	58.79	246.5	630.5
**GR0-4**	579	403.86	58.26	215.1	596.9
**BW6**	599	775.54	118.59	315	1199
**BW8**	598	1147.7	185.55	662	1855.2
**GR4-8**	584	714.04	141.26	205	1258.3
**BW10**	598	1510.34	272.44	760	2419
**BW12**	598	1895.33	365.81	936	3094.5
**GR8-12**	597	747.11	204.32	110.1	1436
**BW14**	588	2090.48	398.21	1079	3127.5
**DW**	579	1789.54	348.56	866.3	2711.4
**AFW**	577	73.12	32.05	0.01	226.35
**EW**	575	1400.41	287.39	641	2158
**LI**	569	114.62	12	82	165
**TG**	254	24.09	22.09	0	98
**CHOL**	265	130.69	22.27	74	185
**LDH**	263	769.08	340.6	336	1729

Supplementary Table 3 Summary of the F_2 GWAS results for all the studied traits

Traits	Nb of SNP (1% significance)	Nb of SNP (5% significance)	peakSNP	Chr.	Location	P value
**BW2**	0	0	rs13974906	1	171,778,431	4.81E-06
**BW4**	6	16	rs13974906	1	171,778,431	1.92E-09
**BW6**	11	18	GGaluGA055431	1	170,802,565	1.95E-09
**BW8**	15	25	GGaluGA055630	1	171,387,660	2.07E-09
**BW10**	24	33	GGaluGA055630	1	171,387,660	1.21E-10
**BW12**	15	27	GGaluGA054960	1	169,679,782	3.16E-09
**BW13**	13	13	GGaluGA055630	1	171,387,660	1.46E-08
**DW**	0	0	rs15243919	27	4,875,779	0.000176822
**AFW**	0	0	rs13972243	1	169,578,430	1.53E-05
**EW**	0	0	rs14120230	19	4,927,473	6.96E-05
**SW**	52	70	rs14917305	1	169,795,686	1.61E-18
**IL**	2	2	GGaluGA054496	1	168,446,325	5.96E-07
**GR0-4**	7	14	rs13974906	1	171,778,431	1.84E-09
**GR4-8**	10	17	GGaluGA054970	1	169,695,275	1.13E-08
**GR8-12**	1	7	rs15501372	1	170,616,617	1.80E-07

Supplementary Table 4 Summary of the F_9 GWAS results for all the studied traits

Traits	Nb of SNP (1% significance)	Nb of SNP (5% significance)	peakSNP	Chr.	Location	P value
**BW2**	10	26	S1_168536487	1	169,241,142	3.84E-12
**BW4**	47	69	S1_168536487	1	169,241,142	6.05E-16
**BW6**	30	54	S1_168536487	1	169,241,142	1.16E-15
**BW8**	54	86	S1_168536487	1	169,241,142	3.38E-16
**BW10**	23	39	S1_168536487	1	169,241,142	2.81E-14
**BW12**	8	17	S1_168536487	1	169,241,142	6.29E-13
**BW14**	4	8	S1_168536487	1	169,241,142	3.67E-12
**DW**	0	0	S4_19243419	4	19,926,211	6.43E-06
**AFW**	0	0	S25_992356	25	1,028,439	2.59E-05
**EW**	0	0	S4_19243419	19	19,926,211	2.16E-06
**IL**	4	9	S1_168536487	1	169,241,142	5.26E-12
**GR0-4**	42	67	S1_168536487	1	169,241,142	7.89E-16
**GR4-8**	15	30	S1_168536487	1	169,241,142	7.04E-12
**GR8-12**	0	0	S3_16954182	3	17,624,830	4.93E-05
**CHO**	0	0	S6_32970253	6	33,486,374	3.28E-05
**TG**	0	0	S3_103616838	3	104,414,933	7.16E-06
**LDH**	0	0	S23_4544444	23	4,607,157	1.41E-05

Supplementary Table 5 Detailed various types of mutation in GGA1: 168.6 -171.7 Mb

Variation Tpes	HQLA	HB
**SNP**	39,707	32,238
**D (delection)**	1,567	1,496
**SI (short insertion)**	1,333	1,269
**CNV (copy number variation)**	10	11
**INV (inversion)**	8	9
**TD (tandem duplication)**	6	4
**Total**	42,631	33,632

Supplementary Table 6. Data statistics for the ATAC-seq

Sample	HBW1	HBW2	LBW1	LBW2
All reads	126444264	142863910	113588079	94890795
Mapped	124822301	140614366	112227552	93381948
Mapped Rate	0.987	0.984	0.988	0.984
Unique Mapped	114594307	131686138	103270285	86853094
Unique Mapped Rate	0.906	0.922	0.909	0.915
All Base	13345901288	14912254164	11059130056	11045461751
Mapped Base	13263443636	14755933973	10989247657	10918951025
Unique Mapped Base	12360940179	13972887791	10242052043	10272456790
Peak Number	29724	21485	19514	7303

Supplementary Table 7. Peak distribution from the ATAC-seq near coding regions

Genome location	HBW1	HBW2	LBW1	LBW2
**Intergenic**	54.93%	53.54%	53.02%	58.46%
**Intron**	25.62%	22.57%	21.97%	24.95%
**Promoter-TSS**	15.93%	21.10%	21.05%	14.97%
**TTS**	1.90%	1.86%	2.06%	1.01%
**Exon**	1.62%	0.93%	1.90%	0.61%

Supplementary Table 8. Allele frequency of miR-16 mutation for the two breeds used as F0 for the intercross line and 10 populations

Cluster	Breeds	Size	Type I (freq.)	Type II (freq.)
High body weight	HQLA (F0)	15	0.37	0.63
	HQLA	25	0.44	0.56
	CB1	29	0.15	0.85
	CB2	30	0.25	0.75
Low body weight	HB (F0)	15	0.25	0.75
	HB	25	0.20	0.80
	SK	30	0.02	0.98
	CH	30	0.39	0.61
	TBC	30	0.45	0.55
	DWS	31	0.02	0.98
	XSD	8	0.13	0.87
	SUM	15	0.08	0.92

Supplementary Table 9. Allele frequency of the rs13553102 SNP in 11 chicken breeds

Cluster	Breeds or lines	Abbreviation	Allele frequency	
			A	G
High body weight	High quality line A03	HQLA	0.78	0.21
	Cobb500	CB1	0.51	0.48
	Recessive white Rock	CB2	0.53	0.46
Low body weight	Huiyang Bearded chicken	HB	0.13	0.86
	Silkies	SK	0.31	0.68
	Chahua chicken	CH	0.25	0.75
	Tibetan chicken	TBC	0.08	0.92
	Daweishan Mini chicken	DWS	0.68	0.31
	Xishuangbanna Game chicken	XSD	0.75	0.25
	Sumatera and Kedu Hitam	SUM	0.53	0.46
	Red jungle fowl	RJF	0.05	0.95

Supplementary Table 10 Primers used for validation of differentially expressed genes

Primer	Sequence
SUCLA2-F	CATGGGTTTCAGGTGCAACA
SUCLA2-R	GAACAGAAATGCCAGCCTCC
NUDT15-F	CTGAAGGGGTACACAGGGAG
NUDT15-R	CGACCCGTCCTTTTGATTCC
MED4-F	GTAATGCTGTTTGTGCCCCT
MED4-R	CATCCCCTGGTAAGTGTCCA
ITM2B-F	ATCCTGCTGACATCGTCCAT
ITM2B-R	CCGGCCTTGATGTTGATGAG
RB1-F	ACAGTAGTACCCGCTGAGGA
RB1-R	GTGCCGTTTGTGGAGGTTTC
LPAR6-F	ATGGTATTTGTCCTCGGCCT
LPAR6-F	ATGTACGTCGTGGTCTCGTT
ENSGALG00000032151-F	TTTCGTTTGCATGCACTTTC
ENSGALG00000032151-R	GTCCCACAAGAGGGTTTCAA
RCBTB2-F	CCTGGATGTGATGGCATTGG
RCBTB2-R	AGTGCCAAGGAAAGTGCATG
FNDC3A-F	TTAACAGCCTTGGAGCAGGA
FNDC3A-R	TCCCCACTTCAGCTTGAGAG
MLNR-F	CCAGGACCTACACAGAGACC
MLNR-R	TGGCAGATGTCCACCTTTCT
CDADC1-F	TTTGGTGAACTGGAGGGTGT
CDADC1-R	TGGTGCTGCTGGTCATCTAT
CAB39L-F	GTCTGCAGTTGTTCTTGCCA
CAB39L-R	GCCTTGCTCCATTGTCACAA
SETDB2-F	TGAAGGCATTTTGGACGCAA
SETDB2-R	TTCGTTCACCAATGCCCAAG
PHF11-F	GGACAGGACTATCAGCAGCT
PHF11-R	GATGCAGACAGAGAAGTGGC
RCBTB1-F	AGGGCGTTACTCCTGTTCAA
RCBTB1-R	CGCCATGGAATGATGAGAGC
ARL11-F	TCCACACTGCTATCCTGGTG
ARL11-R	TCACATCATCCATTGGCATT
KPNA3-F	GCCAGCATTGTGTGTTCTCA
KPNA3-R	TGGTGACTCAGAAGGGGAAC
ENSGALG00000029963-F	CTGAAGCCAGCGAAACTACC
ENSGALG00000029963-R	TCCCTTCCAGACACACTTCC
SPRYD7-F	GGGATGTCCATAGCCTGGTG
SPRYD7-R	GCTGTTGGCTGGTAGTCTGT
TRIM13-F	TGGCGACCATACAAAGCATG
TRIM13-R	TTCCAAGGTATCCAGCCGAG

KCNRG-F	AGTGTGGAACCGACTGCTCT
KCNRG-R	GAGGCGAAATCCATCTTTGA
GAPDH-F	CGATCTGAACTACATGGTTTACATGTT
GAPDH-R	CCCGTTCTCAGCCTTGACA
gga-mir-15a-F	GCGTAGCAGCACATAATGGT
gga-mir-16-1-F	GCGAGTATTAACTGTGCTGCTG
gga-U6-F	CGATACAGAGAAGATTAGCATGG
