# Supplementary material for: Multiple ancestral haplotypes harboring regulatory mutations cumulatively contribute to a QTL affecting chicken growth traits
Source: Commun Biol. 2020 Aug 28;3:472. doi: 10.1038/s42003-020-01199-3 (PMC7455696; doi:10.1038/s42003-020-01199-3)
Supplement: Supplementary file 2 — Description of Additional Supplementary Files [file 42003_2020_1199_MOESM2_ESM.pdf]

Description of Additional Supplementary Files

File Name: **Supplementary Data**
Description the source data file
