# Supplementary material for: Multiple ancestral haplotypes harboring regulatory mutations cumulatively contribute to a QTL affecting chicken growth traits
Source: Commun Biol. 2020 Aug 28;3:472. doi: 10.1038/s42003-020-01199-3 (PMC7455696; doi:10.1038/s42003-020-01199-3)
Supplement: Supplementary file 4 — Reporting Summary [file 42003_2020_1199_MOESM4_ESM.pdf]

nature research

Corresponding author(s): Xiaoxiang Hu, Örjan Carlborg, Dingming Shu

Last updated by author(s): Jul 23, 2020

Reporting Summary

Nature Research wishes to improve the reproducibility of the work that we publish. This form provides structure for consistency and transparency in reporting. For further information on Nature Research policies, see our Editorial Policies and the Editorial Policy Checklist.

Statistics

For all statistical analyses, confirm that the following items are present in the figure legend, table legend, main text, or Methods section.

n/a	Confirmed	
☐	☒	The exact sample size (*n*) for each experimental group/condition, given as a discrete number and unit of measurement
☐	☒	A statement on whether measurements were taken from distinct samples or whether the same sample was measured repeatedly
☐	☒	The statistical test(s) used AND whether they are one- or two-sided<br>*Only common tests should be described solely by name; describe more complex techniques in the Methods section.*
☐	☒	A description of all covariates tested
☐	☒	A description of any assumptions or corrections, such as tests of normality and adjustment for multiple comparisons
☐	☒	A full description of the statistical parameters including central tendency (e.g. means) or other basic estimates (e.g. regression coefficient) AND variation (e.g. standard deviation) or associated estimates of uncertainty (e.g. confidence intervals)
☐	☒	For null hypothesis testing, the test statistic (e.g. *F*, *t*, *r*) with confidence intervals, effect sizes, degrees of freedom and *P* value noted<br>*Give P values as exact values whenever suitable.*
☒	☐	For Bayesian analysis, information on the choice of priors and Markov chain Monte Carlo settings
☐	☒	For hierarchical and complex designs, identification of the appropriate level for tests and full reporting of outcomes
☐	☒	Estimates of effect sizes (e.g. Cohen's *d*, Pearson's *r*), indicating how they were calculated

Our web collection on statistics for biologists contains articles on many of the points above.

Software and code

Policy information about availability of computer code

Data collection	No software was used.
Data analysis	Qualified reads were aligned to the chicken reference genome (Gallus gallus 5.0) with BWA-MEM (version 0.7.10). Initial BAM files were further processed with reordering, sorting and duplicates marking utilizing the Picard (picard-tools-1.56) package followed by base quality recalibration using the BaseRecalibrator tools in the Genome Analysis Toolkit (GenomeAnalysisTK-3.6, GATK). A mixed linear model (MLM) approach was used for the genome-wide association analyses as implemented in the GCTA package (v1.24). A quantile-quantile (Q-Q) plot generated in R (v3.0.2) was used to assess the potential impact of population stratification. A subset of SNPs was obtained by removing one in each pair of SNPs if the LD was greater than 0.4 using the PLINK v1.07. Evolutionary analyses were conducted in MEGA762 based on independent-pairwise SNPs and the tree is drawn by interactive tree of life (iTOL) online tool.

For manuscripts utilizing custom algorithms or software that are central to the research but not yet described in published literature, software must be made available to editors and reviewers. We strongly encourage code deposition in a community repository (e.g. GitHub). See the Nature Research guidelines for submitting code & software for further information.

Data

Policy information about availability of data

All manuscripts must include a data availability statement. This statement should provide the following information, where applicable:

- Accession codes, unique identifiers, or web links for publicly available datasets
- A list of figures that have associated raw data
- A description of any restrictions on data availability

The raw sequencing data cited in 10.1093/molbev/msv071, 10.1186/s12864-016-2652-z, and 10.1371/journal.pone.0179073. Other raw sequence reads have been

deposited in the SRA database (SRA accession: PRJNA547951 and PRJNA647930). Table 1, Figure 1,2,3,5 are associated these raw data. No restrictions on data availability.

Field-specific reporting

Please select the one below that is the best fit for your research. If you are not sure, read the appropriate sections before making your selection.

☒ Life sciences ☐ Behavioural & social sciences ☐ Ecological, evolutionary & environmental sciences

For a reference copy of the document with all sections, see nature.com/documents/nr-reporting-summary-flat.pdf

Life sciences study design

All studies must disclose on these points even when the disclosure is negative.

Sample size	GWAS sample size were 493 for F2 generation and 595 for F9 generation. Population genetics study were employed 12 breeds with sample size of 8 to 31. Nine birds with high 7-week weight and 10 with low 7-week weight were used for the quantitative RT-PCR.
Data exclusions	No data were excluded.
Replication	Replication were successful.
Randomization	Allocation were random.
Blinding	Investigators were not blinded to group allocation.

Reporting for specific materials, systems and methods

We require information from authors about some types of materials, experimental systems and methods used in many studies. Here, indicate whether each material, system or method listed is relevant to your study. If you are not sure if a list item applies to your research, read the appropriate section before selecting a response.

Materials & experimental systems

n/a	Involved in the study	
☒	☐	Antibodies
☒	☐	Eukaryotic cell lines
☒	☐	Palaeontology and archaeology
☐	☒	Animals and other organisms
☒	☐	Human research participants
☒	☐	Clinical data
☒	☐	Dual use research of concern

Methods

n/a	Involved in the study	
☒	☐	ChIP-seq
☒	☐	Flow cytometry
☒	☐	MRI-based neuroimaging

Animals and other organisms

Policy information about studies involving animals; ARRIVE guidelines recommended for reporting animal research

Laboratory animals	No laboratory animals.
Wild animals	No wild animals.
Field-collected samples	A large intercross pedigree was established from two divergent chicken lines, High Quality chicken Line A (HQLA), a broiler line bred by Guangdong Wiz Agricultural Science and Technology, Co. (Guangzhou, China), and Huiyang Bearded chicken (HB), a native Chinese breed.
Ethics oversight	All animals used in this study were cared for, and experiments conducted using procedures, that complied with the requirements of the Animal Welfare Committee of Agrobiotechnology of China Agricultural University (approval SKLAB-2014-06-07).

Note that full information on the approval of the study protocol must also be provided in the manuscript.
